# Polycyclic Aromatic Hydrocarbons from Fine Particulate Matter Induce Oxidative Stress and the Inflammatory Response in Human Vocal Fold Fibroblast Cells

**DOI:** 10.1155/2021/5530390

**Published:** 2021-08-03

**Authors:** Hyunsu Choi, Choung-Soo Kim

**Affiliations:** ^1^Clinical Research Institute, Daejeon St. Mary's Hospital, 64 Daeheung-ro, Jung-gu, Daejeon 301-723, Republic of Korea; ^2^Department of Otolaryngology-Head and Neck Surgery, College of Medicine, The Catholic University of Korea, Daeheung-ro, Jung-gu, Daejeon 301-723, Republic of Korea

## Abstract

Polycyclic aromatic hydrocarbons (PAHs) are toxicants in particulate matter (PM). The vocal fold, part of the larynx and a key structure for voicing, is always in contact with air. In recent epidemic studies, PM was shown to cause laryngitis; however, the basic mechanism has not been evaluated. In the present study, intracellular reactive oxygen species (ROS) and proinflammatory cytokine levels were analyzed after exposing human vocal fold fibroblasts (hVFFs) to PM standard reference material (SRM 2786). Expression levels of the aryl hydrocarbon receptor (AhR) and Cytochrome P450 Family 1 Subfamily A Member 1 (CYP1A1) were also evaluated. PM induced ROS formation and proinflammatory cytokines via the AhR CYP1A1 pathway and caused lipid peroxidation and DNA damage. Blocking AhR or CYP1A1 production using siRNAs significantly decreased ROS production and IL-6 and IL-9 expression in PM-exposed hVFFs, thus protecting the cells against oxidative stress. These results confirm that PAHs in PM play an important role in cell damage and inflammation, confirming a basic pathophysiologic relationship between PM exposure and laryngitis.

## 1. Introduction

Laryngitis generally refers to inflammation of the larynx. There are many causes of laryngitis, including viral or bacterial infection, poor laryngeal hygiene, and laryngopharyngeal reflux [1].

Among these causes, acute laryngitis due to viruses or bacteria is self-limiting and can be treated with appropriate drugs [2]. Chronic laryngitis is caused by poor hygiene or laryngopharyngeal reflux, which can be improved using modified lifestyle or eating habits [3].

If laryngitis is caused by routine breathing, treatment and prevention are difficult.

Air pollution is a major public health problem worldwide. Particulate matter (PM) is a major component of air pollution, which contains numerous organic compounds and inorganic metals. In several epidemiologic studies, PM in air pollution was shown to increase the risks of cardiorespiratory disease and diabetes [4–6]. The effects of PM on systemic and respiratory disease have been widely studied [7, 8]. However, the relationship between laryngitis and PM has been reported in only a few epidemiological studies, and the pathophysiology has not been examined [9–12].

Polycyclic aromatic hydrocarbons (PAHs), also known as aryl hydrocarbon receptor (AhR) ligands, are toxicants in PM. In recent studies, AhR was identified as an important modulator of inflammation [13]. Neavin et al. [14] suggested that a single nucleotide polymorphism near AhR-regulated genes contributes to AhR-related diseases such as asthma and rheumatoid arthritis. Zhu et al. [15] identified the signaling pathway by which AhR promotes interleukin- (IL-) 10 production and suggested that AhR is a potential target for inflammatory disease treatment.

Vocal fibroblasts are important for maintaining the homeostasis of the vocal fold and modify the extracellular matrix when the vocal fold is inflamed or injured by producing various materials in the larynx [16–20]. Therefore, exposing vocal fibroblasts to PM may provide basic information to understand the relationship between PM and laryngitis.

In the present study, the effects of PM, particularly PAHs, on human vocal fold fibroblasts (hVFFs) were investigated with a focus on the inflammatory response and signaling pathways involved.

## 2. Materials and Methods

### 2.1. Reagents

Dulbecco's modified Eagle's medium (DMEM), fetal bovine serum (FBS), and antibiotic-antimycotic solution were obtained from Gibco (Thermo Fisher Scientific, Waltham, MA, USA). The PM standard reference material SRM 2786 was obtained from the National Institute of Standards and Technology (Gaithersburg, MD, USA). 4′,6-Diamidino-2-phenylindole (DAPI) and 2′,7′-dichlorofluorescein diacetate (DCFH-DA) were purchased from Invitrogen (Carlsbad, CA, USA). *N*-acetylcysteine (NAC, an antioxidant) was purchased from Sigma-Aldrich (St. Louis, Mo, USA). Antibodies against AhR and Cytochrome P450 Family 1 Subfamily A Member 1 (CYP1A1) were purchased from Abcam (Cambridge, UK). Antibodies against glyceraldehyde phosphate dehydrogenase (GAPDH) and lamin-B1 were purchased from Cell Signaling Technology (Danvers, MA, USA). Small interfering RNAs (siRNAs) against AhR and CYP1A1, and transfection reagents and kits, were purchased from Santa Cruz Biotechnology (Santa Cruz, CA, USA).

### 2.2. Cell Culture

hVFFs were obtained from the University of Wisconsin (Madison, WI, USA). The cells were grown in culture dishes at 37°C in 5% CO_2_ using DMEM supplemented with 10% FBS and antibiotic-antimycotic solution according to the manufacturer's instructions. The culture medium was replaced every 2 days. Cells were plated at 70–80% confluence and used the next day.

### 2.3. Immunofluorescence Assay

Cultured cells were fixed with 4% paraformaldehyde for 15 min, permeabilized with 0.1% Triton X-100 for 10 min, and blocked with 4% bovine serum albumin for 2 h. Next, the cells were incubated with the primary antibody for 1 h at room temperature (RT). After washing with phosphate-buffered saline (PBS), the cells were incubated with fluorescently labeled secondary antibodies for 30 min at RT. Nuclei were counterstained with DAPI. Slides were mounted and observed under a fluorescence microscope. The following antibodies were used: anti-AhR primary antibodies (Abcam, 1 : 100), anti-4-HNE (Abcam, 1 : 25), and anti-8-OHdG (Abcam, 1 : 200).

### 2.4. Measurement of Intracellular Reactive Oxygen Species (ROS) Levels

A dichloro-dihydro-fluorescein diacetate (DCFH-DA (Invitrogen) fluorescent dye probe was used to measure intracellular ROS production. After different treatments, cells were washed with PBS and then incubated with 10 *μ*M DCFH-DA in PBS at 37°C for 15 min in the dark. The cells were washed twice with PBS, and images were obtained using an inverted light microscope (Eclipse TE300; Nikon, Tokyo, Japan) equipped with a digital camera.

### 2.5. Preparation of Nuclear and Cytoplasmic Protein Extracts

Cytoplasmic and nuclear lysates were separated using a Cell Fractionation Kit (Cell Signaling Technology, Beverly, MA, USA) according to the manufacturer's protocol. Briefly, cultured cells were collected using centrifugation, washed in cold PBS three times, suspended in 500 *μ*L cytoplasm isolation buffer, briefly vortexed, and incubated on ice for 5 min. Next, the cytosolic proteins were pelleted by centrifugation at low speed. The supernatant was collected and stored at –20°C. The pellets were resuspended in 250 *μ*L nucleus isolation buffer. The suspension was incubated on ice for 5 min and sonicated three times for 5 s at 20% vibration. The supernatant containing the nuclear protein extract was transferred to a fresh microcentrifuge tube and stored at –20°C.

### 2.6. siRNA Transfection

Silencing of the genes encoding AhR and CYP1A1 was achieved by transfecting cells with either AhR or CYP1A1 siRNA according to the manufacturer's instructions (Santa Cruz Biotechnology). Briefly, cells (70% confluent) were transfected using Lipofectamine® 2000 (Santa Cruz Biotechnology) for 24 h with either AhR or CYP1A1 siRNA. Then, the cells were washed and incubated with PM for an additional 24 h.

### 2.7. Western Blotting

Total cellular protein from different treatment groups was obtained using RIPA buffer (Elpis Biotech, Daejeon, Republic of Korea) containing protease inhibitor cocktail tablets (Roche Diagnostics, Mannheim, Germany). The protein concentration was measured using a BCA Protein Assay Kit (Pierce, Rockford, IL, USA). Equal amounts of protein (20 *μ*g) were separated by electrophoresis on a 10% sodium dodecyl sulfate-polyacrylamide gel and electrophoretically transferred to a nitrocellulose membrane (Bio-Rad Laboratories, Hercules, CA, USA). Then, the membrane was blocked with 5% nonfat milk in Tris-buffered saline containing 0.05% Tween-20 (TBST buffer) for 1 h and washed three times with TBST buffer for 5 min. Next, the membranes were incubated with different primary antibodies against AhR, CYP1A1, GAPDH, and lamin-B1 at a dilution of 1 : 1000 in 5% nonfat milk in TBST (1 : 1,000) overnight at 4°C. After washing three times in TBST, the PVDF membranes were incubated with anti-mouse or anti-rabbit horseradish peroxidase-conjugated secondary antibodies (1 : 2,000) for 1 h at RT. Positive bands were detected and analyzed using chemiluminescence technology with ChemiDoc™ XRS+ (Bio-Rad Laboratories).

### 2.8. Quantitative Reverse Transcription-PCR (qRT-PCR)

Total RNA was extracted using easy-BLUE™ Total RNA Extraction Kits (iNtRON Biotechnology, Sungnam, Gyeonggi, Korea). Reverse transcription was performed using Reverse Transcriptase Premix (Elpis Biotech). qRT-PCR was performed with an ABI 7500 FAST instrument (Applied Biosystems, Foster City, CA, USA) using GoTaq® Master Mix (Promega, Madison, WI, USA). The gene expression levels were determined by normalizing to GAPDH using the 2^–*ΔΔ*CT^ method. The following primers were used: GAPDH: F, AGCCACATCGCTCAGACAC; R, GCCCAATACGACCAAATCC; CYP1A1: F, TGAACCCCAGGGTACAGAGA; R, GGCCTCCATATAGGGCAGAT; IL-6: F, AACCTGAACCTTCCAAAGATGG; R, TCTGGCTTGTTCCTCACTACT; and IL-8: F, AAGAGAGCTCTGTCTGGACC; R, GATATTCTCTTGGCCCTTGG.

### 2.9. Quantification of IL-6 and IL-8 via Enzyme-Linked Immunosorbent Assay (ELISA)

The protein expression levels of IL-6 and IL-8 in the culture supernatant were measured using BD OptEIA™ ELISA Kits (BD Biosciences, San Jose, CA, USA) according to the manufacturer's instructions. Values were expressed as pg/mL and deduced from standard curves of recombinant cytokines.

### 2.10. Statistical Analysis

The Graph Pad Prism 5 software (GraphPad, Inc., La Jolla, CA, USA) was used to analyze all data. The significance of differences between control and experimental values was assessed using the unpaired *t*-test or one-way analysis of variance (ANOVA). All values are expressed as mean ± standard error of the mean (SEM).

## 3. Results

### 3.1. PM Induces the Upregulation of CYP1A1 Expression by Activating AhR

**Figure 1:**
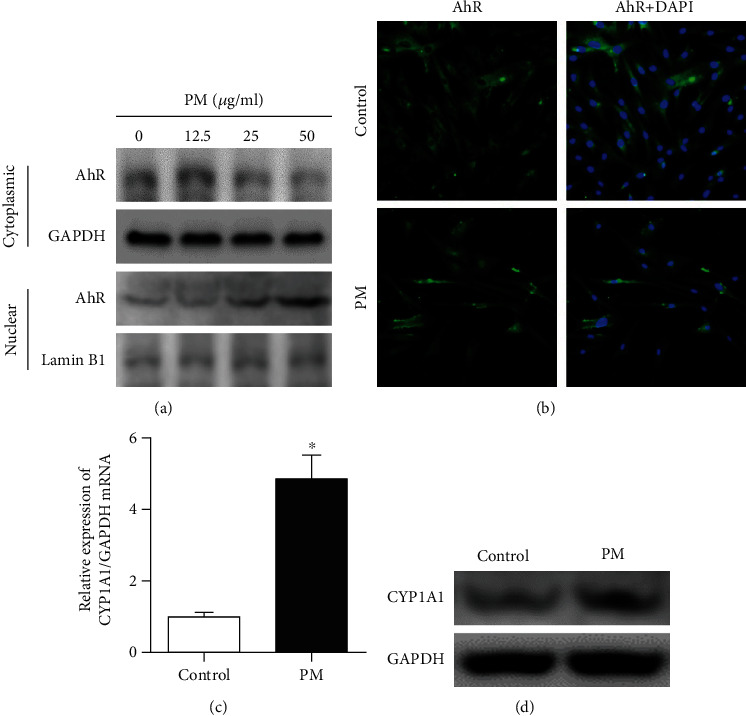
PM induced the nuclear translocation of AhR and upregulated CYP1A1 expression in hVFFs. (a) Nuclear translocation of AhR in hVFFs was analyzed by Western blotting. (b) Nuclear localization of AhR in hVFFs was visualized by immunofluorescence (green, magnification: 200x). Nuclei were stained with DAPI (blue). (c, d) The mRNA and protein levels of CYP1A1, classic target gene of AhR, in hVFFs were measured using Western blotting and qRT-PCR, respectively. ^∗^*p* < 0.05 compared to the control.

To verify whether PM could activate AhR in hVFFs, the effects of PM on AhR nuclear import and CYP1A1 expression in hVFFs were investigated. In addition, the cytoplasmic and nuclear AhR protein levels in hVFFs were evaluated to determine the concentration of PM that affected AhR and upregulated CYP1A1. Panels A and B of Figure 1 show that PM enhanced nuclear translocation of AhR at a concentration of 25 *μ*g/mL. Similarly, PM enhanced the mRNA and protein expression of CYP1A1, indicating that PM activated AhR in hVFFs (Figures 1(c) and 1(d)).

### 3.2. The Effects of PM on hVFFs

**Figure 2:**
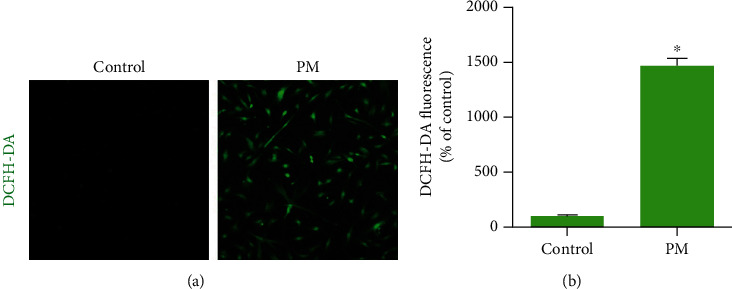
PM-induced ROS generation in hVFFs. (a) After treatment with 25 *μ*g/mL PM for 24 h, the intracellular ROS levels were determined using the DCFH-DA probe by inverted fluorescence microscopy (magnification, 200x). Green color indicates DCF-positive cells. (b) Relative intensity of DCF fluorescence. All experiments were performed in triplicate. Values are the mean ± SEM. ^∗^*p* < 0.05 compared to the control.

**Figure 3:**
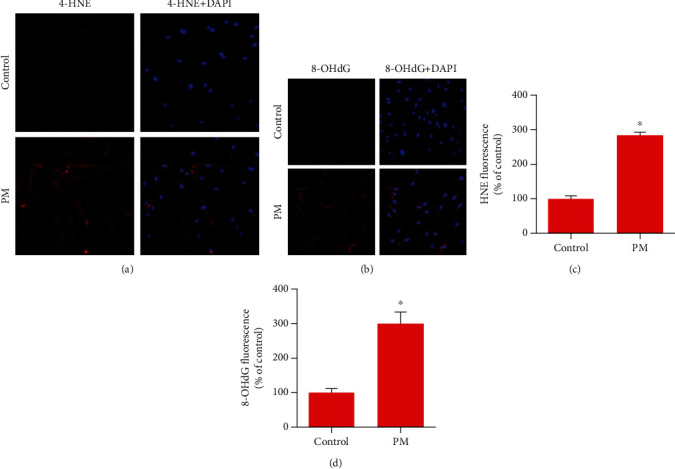
PM induced lipid peroxidation and oxidative DNA damage generation in hVFFs. (a, b) After treatment with 25 *μ*g/mL PM for 24 h, lipid peroxidation was evaluated by 4-HNE immunoreactivity and oxidative DNA damage was evaluated by 8-OHdG immunoreactivity, respectively (red, magnification: 200x). Nuclei were stained with DAPI (blue). (c, d) Quantification of the fluorescence intensities of 4-HNE and 8-OHdG, respectively. All experiments were performed in triplicate. Values are the mean ± SEM. ^∗^*p* < 0.05 compared to the control.

Intracellular ROS production was detected with DCFHA-DA (Figure 2(a)). Densitometry showed that the ROS expression level was increased in hVFFs at a PM concentration of 25 *μ*g/mL and after 24 h of exposure (Figure 2(b)). Next, under the same conditions, oxidative cell damage was evaluated based on lipid peroxidation (4-HNE) and oxidative DNA damage (8-OHdG). Densitometric analysis of 4-HNE and 8-OHdG revealed significant increases compared to control samples (Figure 3).

**Figure 4:**
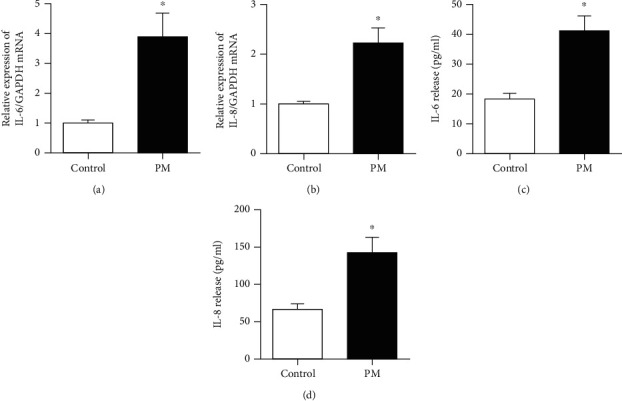
PM upregulated IL-6 and IL-8 expression in hVFFs. (a, b) After treatment with PM (25 *μ*g/mL) for 24 h, mRNA expression of IL-6 and IL-8 was determined by qRT-PCR, respectively. (c, d) Secretion of IL-6 and IL-8 determined by ELISA. All experiments were performed in triplicate. Values are the mean ± SEM. ^∗^*p* < 0.05 compared to the control.

In addition, the relationship between PM and inflammatory cytokines was investigated by evaluating the mRNA expression and protein levels of IL-6 and IL-8. IL-6 and IL-8 expression was significantly increased in hVFFs at a PM concentration of 25 *μ*g/mL after 24 h of exposure compared to the control samples (Figure 4).

### 3.3. PM Increased ROS Formation through the AhR Pathway

**Figure 5:**
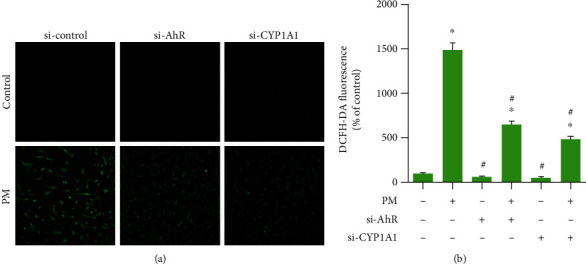
AhR and CYP1A1 knockdown inhibited PM-induced ROS generation in hVFFs transfected with either AhR or CYP1A1 siRNA before treatment for 24 h with PM (25 *μ*g/mL). (a) Intracellular ROS levels were determined using the DCFH-DA probe by inverted fluorescence microscopy (magnification, 200x). Green color indicates DCF-positive cells. (b) Relative intensity of DCF fluorescence. All experiments were performed in triplicate. Values are the mean ± SEM. ^∗^*p* < 0.05 compared to the untreated control and ^#^*p* < 0.05 compared to the PM-treated control.

The effects of AhR and CYP1A1 on ROS formation were investigated by measuring ROS generation induced by FPM after blocking AhR and CYP1A1. Si-AhR and si-CYP1A1 were used to knock down the receptors. ROS generation was significantly decreased compared to the PM group after transfecting si-CYP1A1 or si-AhR; however, there were also significant differences compared to the control group (Figure 5). These results suggest that AhR and CYP1A1 mostly but do not completely regulate ROS generation induced by PM.

**Figure 6:**
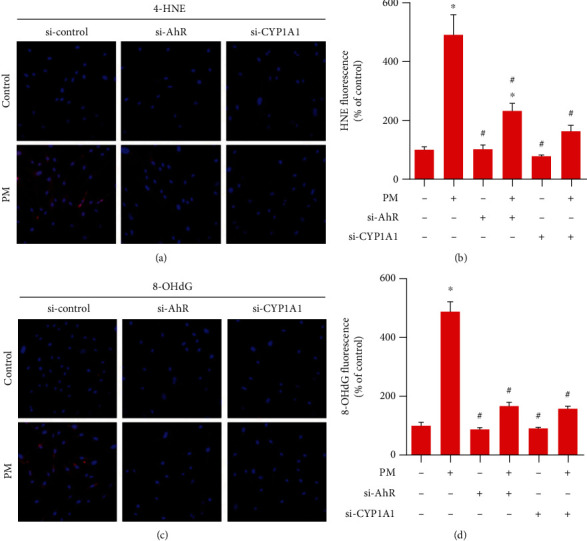
AhR and CYP1A1 knockdown inhibited PM-induced lipid peroxidation and oxidative DNA damage in hVFFs transfected with either AhR or CYP1A1 siRNA before treatment for 24 h with PM (25 *μ*g/mL). (a) Immunofluorescence staining of hVFFs to analyze 4-HNE levels as a marker of lipid peroxidation; signals were detected in a peroxidase reaction (red), nuclei were counterstained with DAPI (magnification, 200x). (b) Fluorescence intensity derived from 4-HNE. (c) The level of 8-oxoG, a hallmark of oxidative DNA damage, was measured based on 8-OHdG detection (red); nuclei were counterstained with DAPI (magnification: ×200). (d) Fluorescence intensity derived from 8-OHdG. All experiments were performed in triplicate. Values are the mean ± SEM. ^∗^*p* < 0.05 compared to the untreated control and ^#^*p* < 0.05 compared to the PM-treated control.

Lipid peroxidation (4-HNE) and oxidative DNA damage (8-OHdG) were investigated before and after transfection of si-AhR or si-CYP1A1 to evaluate oxidative cell damage. Both 4-HNE and 8-OHdG induced by PM were significantly decreased after transfection of si-AhR or si-CYP1A1. However, si-AhR transfection did not reduce lipid peroxidation to control levels. In the 8-OHdG evaluation, si-AhR or si-CYP1A1 transfection reduced oxidative DNA damage to control levels (Figure 6).

### 3.4. AhR and CYP1A1 Participate in the Induction of Proinflammatory Cytokines through ROS by PM

**Figure 7:**
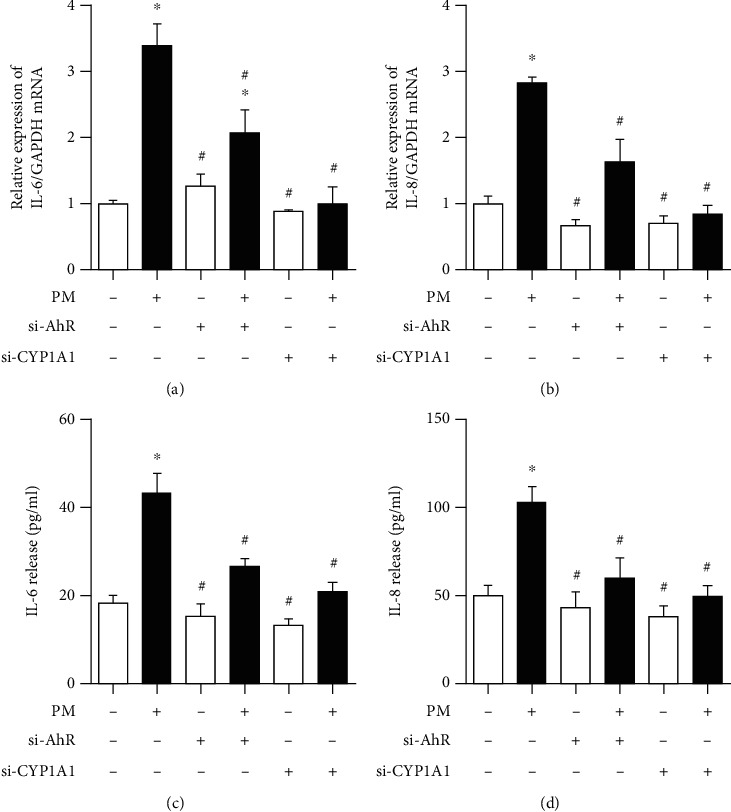
Effects of AhR and CYP1A1 silencing on PM-induced inflammatory cytokines in hVFFs transfected with either AhR or CYP1A1 siRNA before treatment for 24 h with PM (25 *μ*g/mL). (a, b) IL-6 and IL-8 mRNA expression was quantified by qRT-PCR normalized to the GAPDH housekeeping gene. (c, d) The protein levels of IL-6 and IL-8 were measured using ELISA. All experiments were performed in triplicate. Values are the mean ± SEM. ^∗^*p* < 0.05 compared to the untreated control and ^#^*p* < 0.05 compared to the PM-treated control.

**Figure 8:**
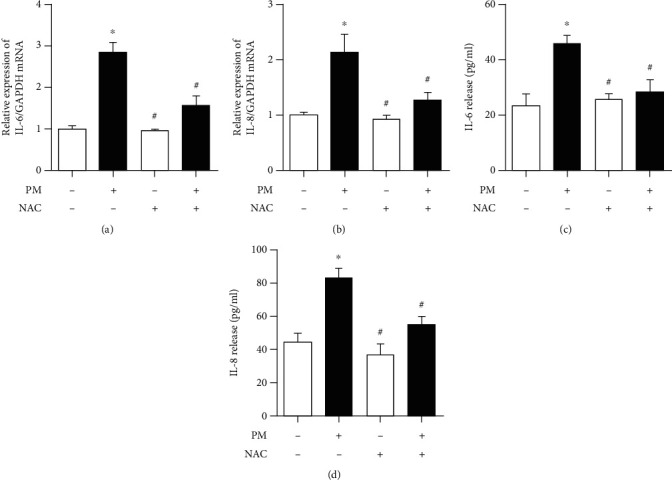
Inhibition of PM-induced IL-6 and IL-8 production by NAC in hVFFs pretreated with or without NAC (1 mM) for 2 h before exposure to PM for 24 h. After incubation, the cells were collected to analyze IL-6 and IL-8 gene expression by qRT-PCR and analysis of cell supernatants for IL-6 and IL-8 protein expression by ELISA. (a, b) Effects of NAC on IL-6 and IL-8 mRNA expression. (c, d) Effects of NAC on IL-6 and IL-8 secretion. Results of three separate experiments are displayed in graphs and are expressed as the mean ± SEM. ^∗^*p* < 0.05 compared to the untreated control and ^#^*p* < 0.05 compared to PM without NAC.

To evaluate the roles of AhR and CYP1A1 in inflammation, IL-6 and IL-8 levels were investigated before and after blocking each. After blocking either, the IL-6 and IL-8 mRNA and protein levels showed that the PM-induced proinflammatory response was significantly decreased compared to the PM group. However, the IL-6 mRNA level was not decreased to the level in the controls (Figure 7). Pretreatment of hVFFS with NAC before exposure to PM with NAC, an inhibitor of ROS, led to significantly decreased IL-6 and IL-8 mRNA and protein levels compared to untreated hVFFs (Figure 8).

## 4. Discussion

In the present study, PM induced ROS production, increased inflammatory cytokines such as IL-6 and IL-8, and damaged hVFFs. These results were mediated by the AhR-CYP1A1 pathway. Inhibition of AhR decreased PM-induced ROS production and effectively protected against ROS-induced cell damage. Furthermore, the expression levels of inflammatory cytokines increased by PM were also significantly decreased. The results showing that the ROS inhibitor significantly reduced inflammatory cytokines indicates that ROS induced an inflammatory response in hVFFs. Therefore, AhR-CYP1A1-ROS formation is a potential mechanism of cell damage and an inflammatory response in hVFFs and may be one mechanism underlying chronic laryngitis.

Vocal fibroblasts are located in the lamina propria of the vocal mucosa. Their role is not fully understood. However, many studies have revealed that these fibroblasts are essential for tissue homeostasis by producing ECM molecules and could alter their function when the vocal fold is damaged. Gugatcschka et al. [21] analyzed the proteomics of vocal fold fibroblasts exposed to cigarette smoke and suggested the altered function of vocal fibroblasts as a key pathogenesis of Reinke's edema. In addition, the vocal folds are anatomic structures that vibrate for vocalization. Therefore, some degree of vocal trauma cannot be avoided compared to other structures in the airway that do not need to vibrate, and under traumatic conditions inflammatory cytokines are secreted [22, 23]. Under normal circumstances, the vocal trauma occurring during vocal fold vibration naturally recovers; however, when an external inflammatory substance is encountered, the vocal fold is inevitably vulnerable to inflammation. Laryngitis usually presents as a hoarse voice, globus sensation, and vocal fold erythema and edema. In previous epidemiological studies, laryngitis was associated with PM. In a large cohort study by Joo et al. [9], increased PM exposure was associated with a higher risk for chronic laryngitis, and PM was equally associated with laryngitis (odds ratio [OR], 1.378) as with smoking (OR, 1.483). Unlu et al. [24] reported that exposure to cigarette smoke induced CYP1A1 expression in vocal folds. However, the underlying mechanism linking laryngitis and PM, particularly PAHs, has not been investigated to date. In the present study, we clarified that PAHs in PM induced ROS production, damaged cells through oxidative stress, and consequently increased inflammatory cytokine levels via the AhR-CYP1A1 pathway.

PAHs are considered to be the most toxic organic chemical in PM. The toxic mechanisms of PAH are through classic and non-classic pathways [25]. Recently, the diversity of nonclassic pathways has been identified, including crosstalk with other nuclear proteins or signaling factors such as nuclear factor kappa B [26]. However, most of the effects of PAH are via the classic pathway. Some studies in transgenic mice with AhR knockout have shown that biological toxicity is through the classic AhR pathway [27, 28]. In this pathway, activated AhR and AhR-dependent CYP1A1 produce ROS, which damages the cell and triggers inflammation [29].

In the present study, si-AhR or si-CYP1A1 did not completely inhibit ROS production. This might be due to other components in PM (e.g., heavy metals) that also produce ROS [30, 31]. Another possible reason is that other P450 enzymes such as CYP1A2, CYP3A1, or CYP2B1 could also produce ROS [32, 33]. Similar results have also been found between si-AhR and si-CYP1A1 and the inflammatory cytokines IL-6 and IL-8. These results are consistent with previous studies in which proinflammatory cytokines were associated with ROS formation [34, 35]. In this study, we also confirmed that proinflammatory cytokines were induced by ROS production, as the mRNA and protein expression levels of proinflammatory cytokines were significantly reduced by NAC in PM-treated hVFFs.

Notably, the protective effects of si-AhR are insufficient to prevent cellular damage due to lipid peroxidation. However, si-AhR sufficiently prevented oxidative DNA damage, indicating that among the components of PM PAHs play an important role in DNA damage via ROS production.

The present study had several limitations. The effects of other PM components were not evaluated. Heavy metals also produce ROS and cause inflammatory responses. Additional studies are needed to investigate the precise effects and underlying mechanisms whereby PM affects the vocal fold. Another limitation is that the exposure time for PM was relatively short; thus, additional studies with longer PM exposure times or animal experiments are necessary.

PM induced ROS production and consequently a proinflammatory response via CYP1A1 in hVFFs. PAH played a major role in the response via the AhR-CYP1A1 pathway. Our results will further our understanding of the basic pathophysiology between PM exposure and laryngitis.

## Data Availability

All data used to support this study are included within the article as references.
